# Genomic analysis of 10 years of artificial selection in community‐based breeding programs in two Ethiopian indigenous sheep breeds

**DOI:** 10.1111/age.13190

**Published:** 2022-04-15

**Authors:** Emna Rekik, Abulgasim M. Ahbara, Zelalem Abate, Shenkute Goshme, Tesfaye Getachew, Aynalem Haile, Barbara Rischkowsky, Joram M. Mwacharo

**Affiliations:** ^1^ Small Ruminant Genomics International Centre for Agricultural Research in the Dry areas (ICARDA) Addis Ababa Ethiopia; ^2^ Department of Zoology Faculty of Sciences Misurata University Misurata Libya; ^3^ Animal Sciences Case Team Bonga Agricultural Research Center Bonga Ethiopia; ^4^ Debre‐Birhan Agricultural Research Center Debre‐Birhan Ethiopia; ^5^ Animal and Veterinary Sciences Scotland Rural College and Centre for Tropical Livestock Genetics and Health (CTLGH) The Roslin Institute Building Easter Bush Midlothian UK

**Keywords:** Bonga, breeds, diversity, genetic improvement, genome‐wide| Menz, *Ovis aries*, selection signatures

## Abstract

In recent times, community‐based breeding programs (CBBPs) have been advocated as the best strategy for genetic improvement of local breeds in smallholder farms in developing countries. Since 2009, CBBPs have been implemented for Ethiopian Bonga and Menz sheep to improve growth rates resulting in significant genetic gains in 6‐month weights. With the hypothesis that selection could be impacting their genomes, we systematically screened for possible genome changes in the two breeds by analyzing 600K BeadChip genotype data of 151 individuals (with the highest breeding values for 6‐month weights) from CBBP flocks against 98 individuals from non‐CBBP flocks. We observed no differences in genetic diversity and demographic dynamics between CBBP and non‐CBBP flocks. Selection signature analysis employing ROH, logistic regression genome‐wide association study , *F*_ST_, XP‐EHH and iHS revealed 5 (Bonga) and 11 (Menz) overlapping regions under selection, that co‐localized with QTLs for production (body size/weight, growth, milk yield), meat/milk quality, and health/parasite resistance, suggesting that the decade‐long selection has likely started to impact their genomes. However, genome‐wide genetic differentiation between the CBBP and non‐CBBP flocks is not yet clearly evident.

Advances in genomics have broadened our capacity to investigate genetic profiles of organisms and the effects of demographic and selection events. Studies analyzing genomic data (SNP genotypes and whole‐genome sequences) are revealing insights on genetic differentiation and the outcomes of selection for aesthetic, adaptive, and economic traits in domestic animals. Such studies have employed two analytical strategies: (i) inter‐ and intra‐breed comparison of haplotypes and allele frequencies (Fu et al., 2016; Naderi et al., 2020; Niu et al., 2021; Tao et al., 2021; Yang et al., 2021); and (ii) intra‐breed analysis exploring patterns of linkage disequilibrium (LD), runs of homozygosity (ROH) and haplotypes associated with selection sweeps (Al‐Mamun et al., 2015; Qanbari et al., 2010, 2011). The first approach reveals differentially fixed loci, and the second detects changes in haplotype frequencies (Qanbari et al., 2011).

Community‐based breeding programs (CBBPs) are owned and run by small‐scale farmers who undertake animal identification, performance and pedigree recording, and systematic selection in their flocks (Mueller et al., 2015; Wurzinger et al., 2011). Typically, the farmers prioritize breeding objectives and selection targets as a community, which they then pursue in small‐scale one‐ or two‐tier breeding structures (Haile et al., 2020). CBBPs have been implemented in Africa (Burkina Faso, Ethiopia, Malawi, Sudan, Tanzania, Uganda), South and Central America (Argentina, Bolivia, Mexico, Peru), and Asia (Iran) for different livestock species (Wurzinger et al., 2008, 2013; Mueller et al., 2015; Haile et al., 2020; Ouédraogo et al., 2020; Kaumbata et al., 2021). Several aspects of CBBPs have been investigated including livestock keepers’ selection criteria and breeding goals, and simulation modelling of potential genetic gains for diverse traits (see Wurzinger et al. [2021] and references therein). However, what is yet to be investigated is the possible effects of farmer‐driven selection on the genomes of CBBP animals.

The analysis of 10 years (2009–2018) of performance data shows that CBBPs are technically feasible, result in measurable genetic gains, and impact the livelihoods of resource poor smallholder farmers (Haile et al., 2020). The analysis (Haile et al., 2020) showed substantial and significant (*p* < 0.05) gains of 0.21 ± 0.018 and 0.11 ± 0.003 kg/year in 6‐month weights in Bonga and Menz sheep from Ethiopia respectively under CBBPs in smallholder farms where resources are limiting. These gains led to the hypothesis that the decade long artificial selection could be impacting the genomes of the CBBP flocks. To test this hypothesis, whole blood was collected from CBBP and non‐CBBP flocks through jugular venipuncture with the K_2_EDTA spray‐coated BD Vacutainer^®^ tubes (Becton, Dickinson and Company, NJ, USA). Genomic DNA was extracted with the DNeasy Blood and Tissue Kit (Qiagen LLC) from 120 and 46 blood samples of individuals, born between 2014 and 2020 in Bonga CBBPs and between 2011 and 2019 in Menz CBBPs respectively. These animals reported the highest estimated breeding values (EBV > 0.22 (Bonga) and >0.11 (Menz)) for 6‐months weight, the primary target selection trait. From non‐CBBP flocks not exposed to artificial selection, DNA extracted from blood samples of 67 (Bonga) and 42 (Menz) individuals of random age were used. The aim was to obtain contrasting genotypes to test for structural evidence of the effects of selection on the genomes of the CBBP flocks. We genotyped the DNA samples with the Ovine 600K SNP BeadChip (Illumina Inc., San Diego, CA, USA) at GeneSeek Neogen Genomics (Lincoln, NE, USA). The BeadChip contains 606,006 probes that target genome‐wide SNPs, among which 577 401 are autosomal, 27 314 are on the X chromosome, and 1291 remain unassigned. Following genotyping, the SNP positions were called referencing the Oar_v3.1 sheep genome assembly and samples with calling rates lower than 90% and markers that remain unassigned were removed. Markers with: (i) missing genotype at most 10%; (ii) Hardy–Weinberg equilibrium exact test *p*‐value above 10^−6^; and (iii) minor allele frequency of at least 0.05, were retained. These quality control filter thresholds were applied across the whole dataset. Consequently, 111 (Bonga) and 40 (Menz) CBBP individuals and 60 (Bonga) and 38 (Menz) non‐CBBP samples, and 494,330 markers were retained for diversity estimates and population structure analysis.

To describe genetic diversity, we calculated expected (*H*_E_) and observed (*H*_O_) heterozygosity, and genomic inbreeding (*F*_ROH_) using PLINK 1.9 (Purcell et al., 2007). The *F*_ROH_ was calculated following Ahbara et al. (2021). No significant differences were observed between CBBP and non‐CBBP flocks for these statistics (Table 1). But the obtained values are within the range of global sheep diversity (Kijas et al., 2012; Zhang et al., 2019). They indicate that the CBBP flocks are still diverse with low levels of inbreeding; they are still reservoirs of indigenous genomic diversity.

**TABLE 1:** Indicators of genetic diversity and measures of runs of homozygosity (ROH) for different classes of animals analyzed in this study

	CBG	NBG	CMZ	NMZ
Sample size	111	60	40	38
*H*_O_	0.3086 ± 0.0116	0.3136 ± 0.0145	0.3270 ± 0.0210	0.3278 ± 0.0207
*H*_E_	0.3114 ± 0.0001	0.3171 ± 0.0001	0.3364 ± 0.0001	0.3373 ± 0.0001
ROH (Mean (Mb) ± SD)	1.716 ± 0.1581	1.953 ± 0.2570	2.625 ± 0.5347	2.585 ± 0.4273
*F*_ROH_ (Mean ± SD)	0.0345 ± 0.0343	0.0404 ± 0.0421	0.0495 ± 0.0599	0.0496 ± 0.0578
Mean number of ROH/animal (range)				
0–5	47.98 ± 25.54 (1–212)	49.98 ± 17.43 (29–150)	40.48 ± 22.89 (1–108)	40.89 ± 21.89 (24–129)
5–10	5.12 ± 7.77 (1–29)	4.60 ± 7.64 (1–27)	8.17 ± 10.15 (1–30)	7.78 ± 9.54 (1–41)
>10	2.44 ± 1.74 (1–6)	5.80 ± 6.14 (1–13)	3.00 ± 2.71 (1–9)	3.00 ± 2.49 (1–9)
Mean length (Mb) of ROH/animal (range)				
0–5	1.49 ± 0.17 (1.08–2.25)	1.54 ± 0.22 (1.29–2.43)	1.70 ± 0.36 (1.01–2.66)	1.75 ± 0.33 (1.25–2.64)
5–10	6.12 ± 0.86 (5.18–8.78)	6.29 ± 0.88 (5.07–8.30)	6.77 ± 1.03 (5.04–9.34)	6.76 ± 0.84 (5.04–8.13)
>10	12.97 ± 3.58 (10.24–22.44)	13.88 ± 4.68 (10.38–22.99)	14.04 ± 4.01 (10.93 ± 26.45)	12.87 ± 1.37 (10.70–15.25)

Abbreviations: CBG, Bonga community‐based breeding program (CBBP) flock; NBG, Bonga non‐CBBP flock; CMZ, Menz CBBP flock; NMZ, Menz non‐CBBP flock; *H*_E_, expected heterozygosity; *H*_O_, observed heterozygosity; *F*_ROH_, genomic inbreeding coefficient.

Demographic dynamics were investigated by exploring ROH at different genomic distance intervals, LD decay patterns and effective population sizes (*N*_E_). Default parameters of *detectRUNS* R package (Biscarini et al., 2019) were used to detect ROH, and LD patterns with PLINK 1.9, while *N*_E_ was calculated following Sved (1971). The average length and number of ROH per individual were assessed at three genomic distance classes (1–5, 5–20, >20 Mb) indicating ancestral (>10 generations), middle (5–10 generations), and recent (<5 generations) inbreeding respectively (Mastrangelo et al., 2018). Both CBBP and non‐CBBP flocks harbor high frequencies of ROH in the 1–5 Mb category (Table 1) reflecting past/ancestral inbreeding. This suggests that the CBBPs are structured well to avoid recent inbreeding through mating management via ram rotation. The CBBP and non‐CBBP flocks show similar patterns of decay in LD (Figure 1a), which mirrors the patterns observed in other sheep populations (Kijas et al., 2014). The Bonga CBBP shows lower LD (*r* ^2^) than its non‐CBBP cohort. The trends in *N*_E_ are the same irrespective of breed or CBBP flock and increases from 1000 up to about 500 generations ago, and then declines to the present (Figure 1b). This suggests common past and recent demographic dynamics independent of the implementation of the CBBPs. Furthermore, the Bonga CBBP shows higher *N*_E_ across all generations compared to its non‐CBBP cohort and the *N*_E_ for Menz CBBP and non‐CBBP flocks does not differ. These point to, as yet, no effect of selection on the *N*_E_ of the CBBP flocks.

**FIGURE 1:**
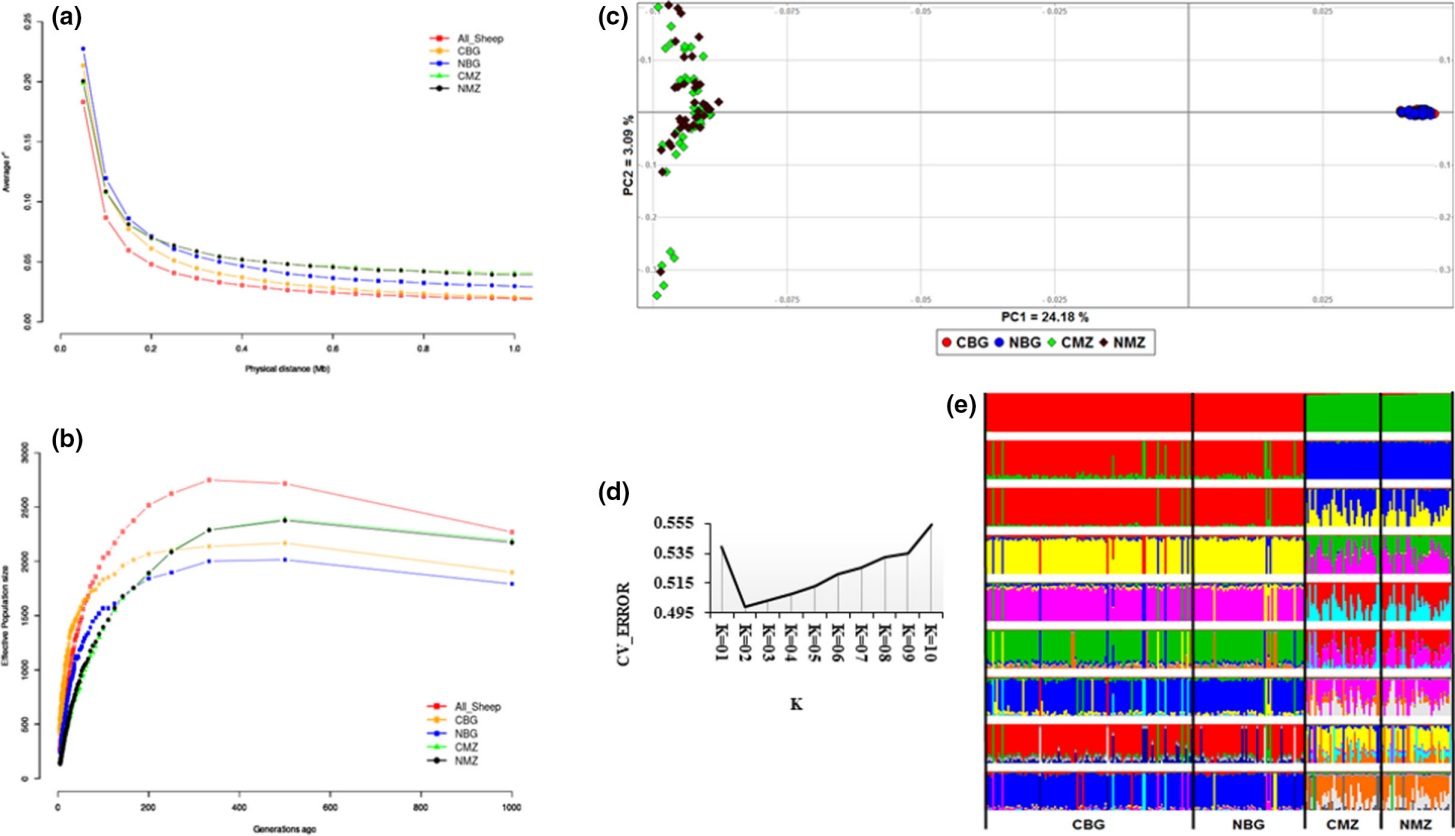
Trends in demographic dynamics depicted by (a) linkage disequilibrium decay over genomic distances and (b) changes in effective population sizes over generation time. Population structure analysis as revealed by (c) Principal component analysis, (d) CV error plot following ADMIXTURE, and (e) ADMIXTURE plot showing assignment probabilities of community‐based breeding program (CBBP) and non‐CBBP flocks from Bonga and Menz. Abbreviations: CBG, Bonga CBBP flock; NBG, Bonga non‐CBBP flock; CMZ, Menz CBBP flock; NMZ, Menz non‐CBBP flock

To explore genetic structure and investigate whether the CBBP flocks are diverging from their non‐CBBP cohorts, we performed principal component (PCA) and ADMIXTURE (Alexander et al., 2009) analyses. The PCA separates Bonga from Menz, as the principal stratification, and not the CBBP and non‐CBBP flocks (Figure 1c). This is supported by ADMIXTURE analysis, which showed that the lowest CV error was at *K* = 2 (Figure 1d) suggesting the presence of two genetic groups, comprising Bonga and Menz sheep (Figure 1e). The separation between Bonga and Menz confirms previous findings (Ahbara et al., 2019). To investigate whether the divergence between Bonga and Menz is masking the divergence between CBBP and non‐CBBP flocks, we performed separate PCAs for each breed. It resulted in no stratification between the cohorts (Figure S1). We hypothesize that this lack of stratification indicates that the effects of selection across the genomes of the CBBP flocks is minimal but could be localized on specific genomic regions. It may also imply that the selection in the CBBP flocks is either not very stringent or that the decade long selection is yet to result in genome‐wide intra‐breed divergence. This lack of genetic differentiation is unsurprising; it has been reported in selected lines of dual‐purpose black and white, and German Holstein cattle (Naderi et al., 2020), between endoparasite‐resistant and ‐succeptible Tunisian indigenous sheep (Ahbara et al., 2021), and between prolific and non‐prolific cohorts of Bonga sheep (Dolebo et al., 2019).

To determine whether the decade‐long selection is imprinting on specific genomic locations of the CBBP *vis*‐*à*‐*vis* non‐CBBP flocks, we investigated selection signatures using ROH, logistic regression genome‐wide association study (LR‐GWAS), *F*_ST_, XP‐EHH, and iHS (Figures S2–S4). ROH and LR‐GWAS were implemented with PLINK, *F*_ST_ with HIERFSTAT (Goudet, 2005), and both iHS and XP‐EHH with rehh (Gautier & Vitalis, 2012). Candidate regions that overlapped between the four methods were identified and merged with BedTools v.2.28.0 (Quinlan & Hall, 2010). There were five (Bonga) and 11 (Menz) candidate regions that overlapped between at least two methods (Table S1), suggesting them to be possible high confidence selection targets. We limited our annotation analysis to these five and 11 candidate regions to search for the presence of known ovine genes and QTLs to predict their functional significance. The NCBI genome (*Ovis aries*, assembly OAR v3.1, NCBI annotation release 101) database was cross‐referenced to detect putative genes.

The five and 11 regions spanned 12 and 30 genes each (Table S1). Over‐representation analysis of gene ontology (GO) terms was done with DAVID 2021 (https://david.ncifcrf.gov) using the genes (12 and 30) in the overlapping candidate regions. The analysis showed that Bonga has increased GO biological process terms (corrected *p*‐value <0.05) involved in innate immune response (GO:0045087) and actin cytoskeleton organization (GO:0030036; Table S2a). In Menz, three significant (corrected *p*‐value <0.05) GO biological process terms, cGMP biosynthetic process (GO:0006182), phospholipid catabolic process (GO:0009395), and positive regulation of I‐κB kinase/NF‐κB signaling (GO:0043123) were enriched (Table S2b). This suggests that the regions are enriched in genes contributing to functions not relating to the breeding goal traits but include regulation of immune (innate and adaptive) response to infectious diseases, regulation of multiple aspects of cellular behaviour, including the control of cell morphology, motility and response to stimuli, and energy biosynthesis.

To further investigate the overlapping selection regions and their possible association with the breeding goal traits, we performed annotation analysis searching for the presence of known ovine QTLs by cross‐referencing the sheep QTLdb (Animal QTL Database [animalgenome.org], Release 44; accessed 16 August 2021) database based on the Oar_v3.1 sheep genome build. It revealed the 5 and 11 regions co‐localized with QTLs for meat/carcass quality/composition, milk quality/composition, body size/weight/growth, and health/parasite resistance, and two QTLs each for reproductive seasonality and testes weight in Menz (Tables S3a,b). One region in Bonga and another in Menz spanned no QTLs. The co‐localization of the candidate regions with some QTLs of economic significance aligns well with the primary selection target (6‐months weight) in the two CBBP flocks, and our comparative analysis that contrasted CBBP and non‐CBBP animals. The results also suggest a correlated selection response for milk yield and quality. This may be due to the increased demand for milk to sustain accelerated growths in the selected animals. It should be noted that the 6‐months weight corresponds to the weaning weight and time for the animals. The overlap with QTLs for health/parasite resistance is surprising as it was not a selection goal trait. However, it is known that endoparasites can limit host nutrient availability by reducing host food intake, digestion, absorption, and nutrient assimilation, resulting in nutritional deprivation and destabilization of growth and development (Goater et al., 1993; Hansen & Perry, 1994). Thus, concurrent indirect selection for endoparasite QTLs may be ensuring growth and development stability in the selected animals under natural endoparasite challenge.

In conclusion, our findings offer the first insights enriching our understanding of the possible genomic effects of artificial selection in CBBPs flocks. Further genomic analysis of subsequent generations will likely reveal deeper insights on the effects of artificial selection, such as the rate and intensity of genomic changes in CBBP flocks in different livestock species.

## CONFLICT OF INTEREST

The authors declare no conflict of interest.

## Supporting information

Figures S1–S4

Table S1–S3

## Data Availability

https://figshare.com/s/d5bce8d70d0d8496d9d6; DOI: d10.6084/m9.figshare.17087663.
