# Supplementary material for: Genomic analysis of 10 years of artificial selection in community‐based breeding programs in two Ethiopian indigenous sheep breeds
Source: Anim Genet. 2022 Apr 15;53(3):447–51. doi: 10.1111/age.13190 (PMC10138745; doi:10.1111/age.13190)

Supplementary Figure S1. PCA Plots

a) Bonga sheep

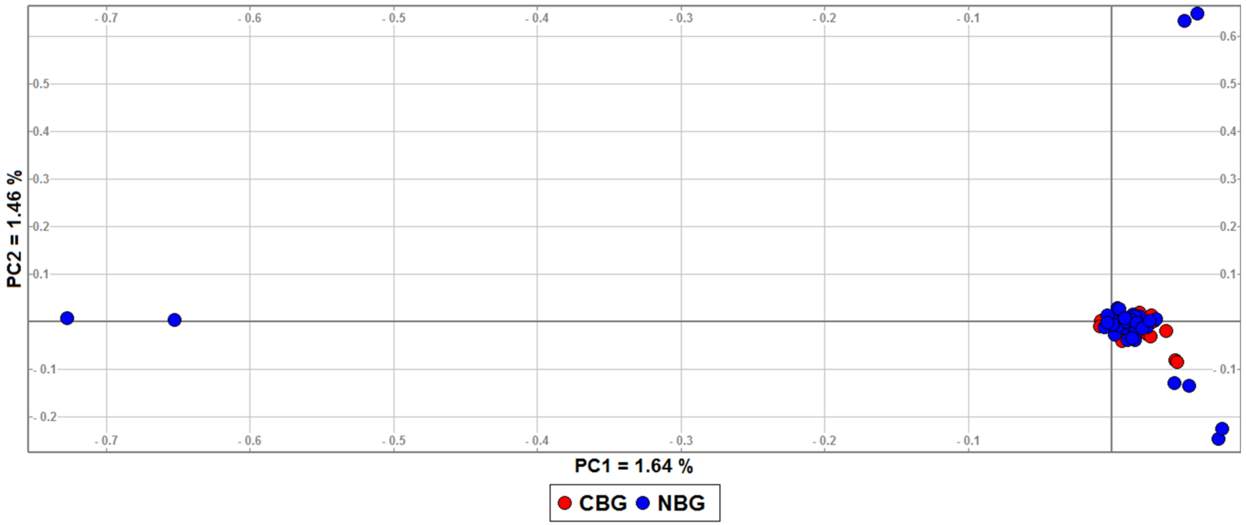

b) Menz sheep

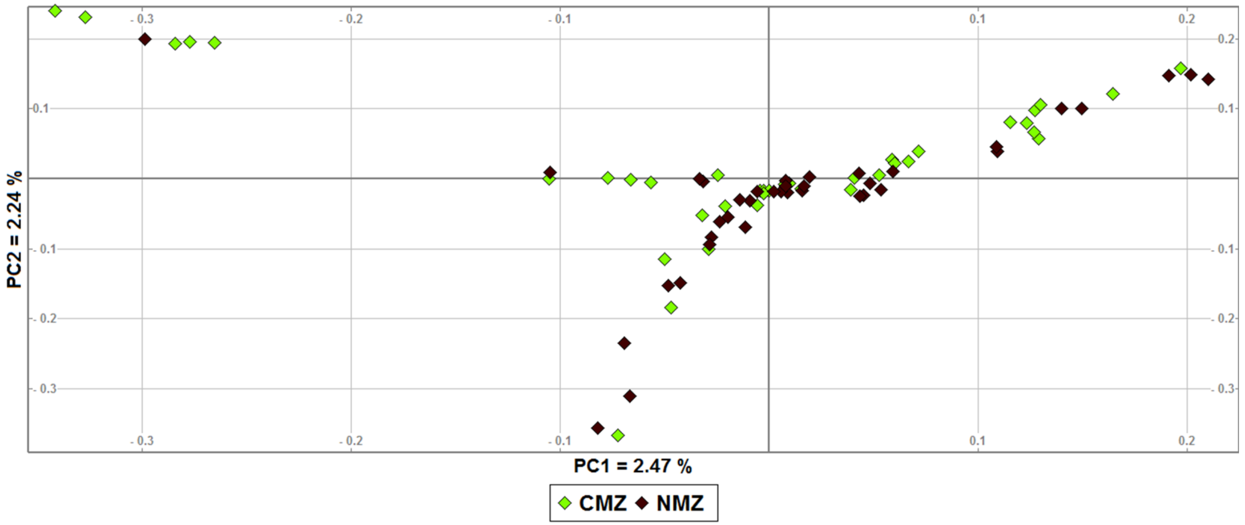

Supplementary Figure S2. ROH

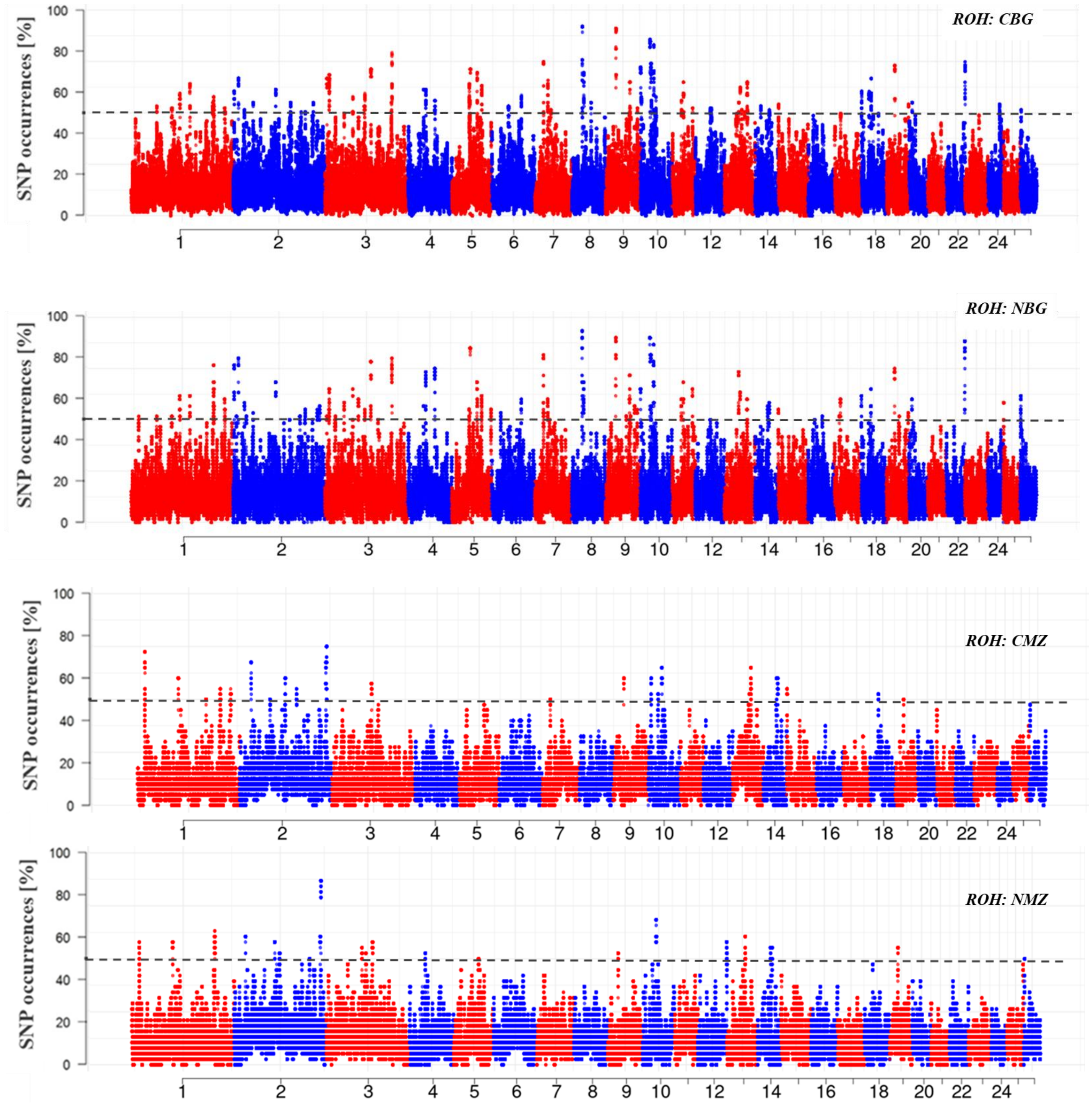

Supplementary Figure S3. LR-GWAS, F_{ST}, XP-EHH

a) Bonga

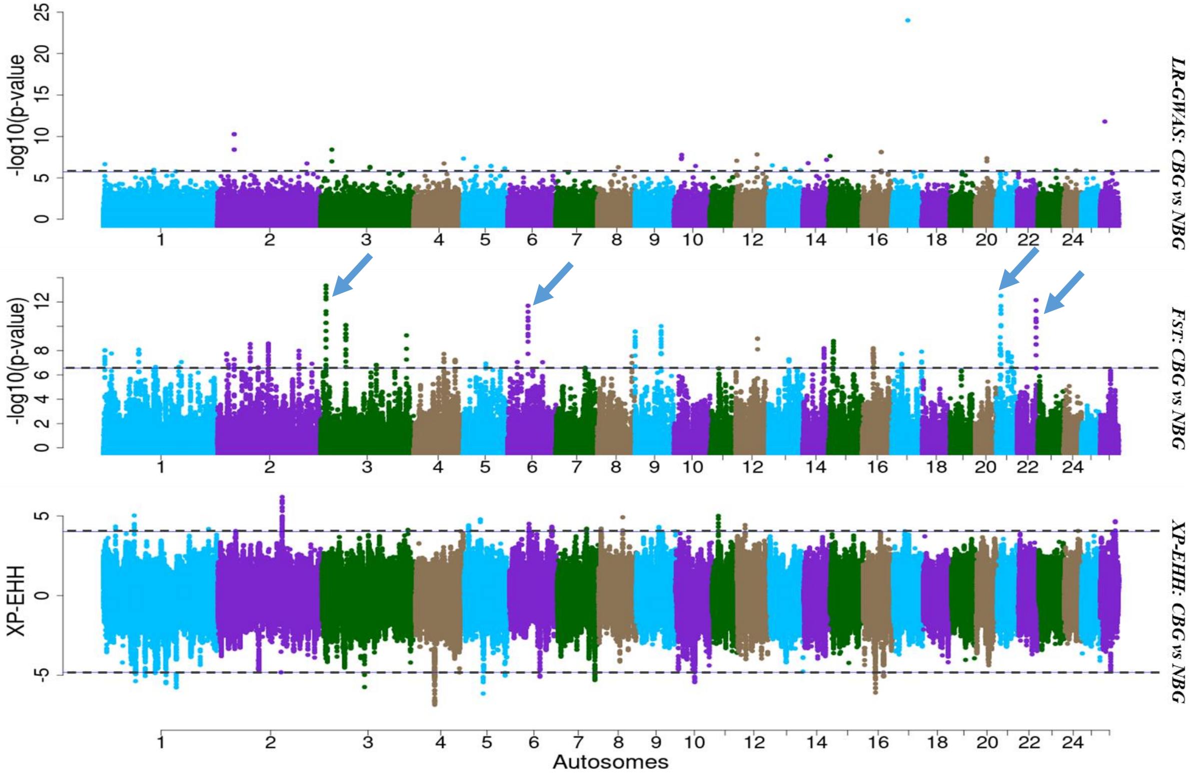

a) Menz

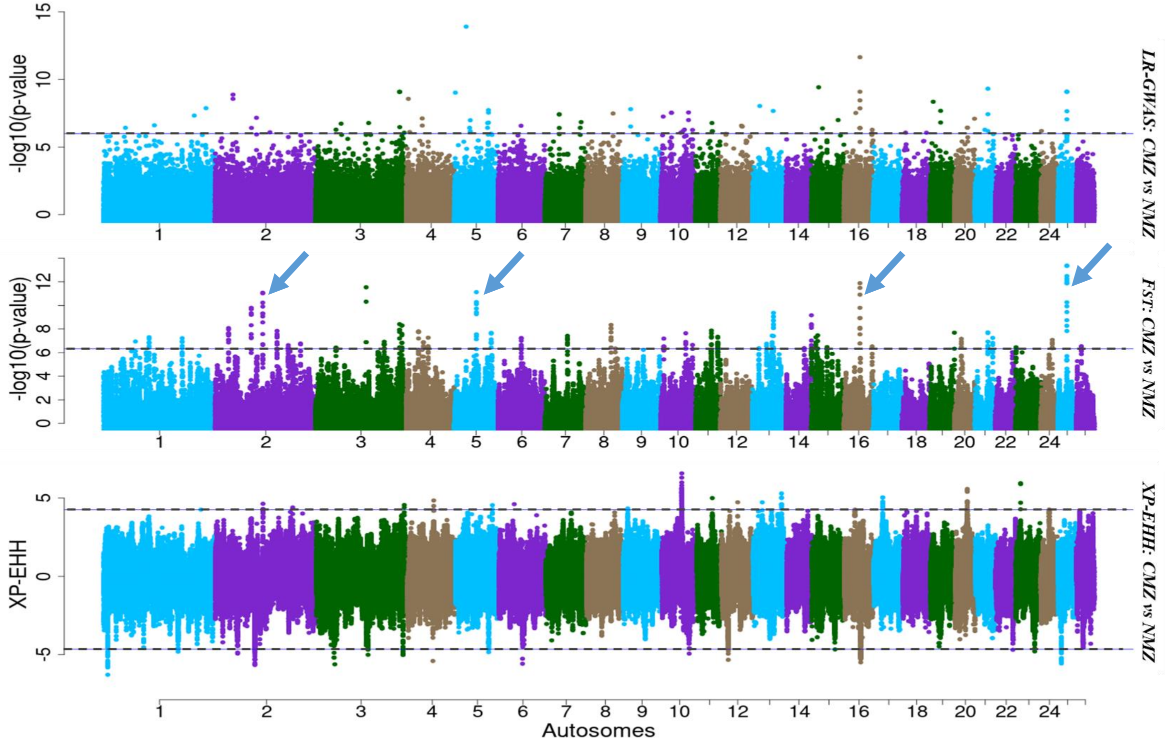

Supplementary Figure S4. iHS

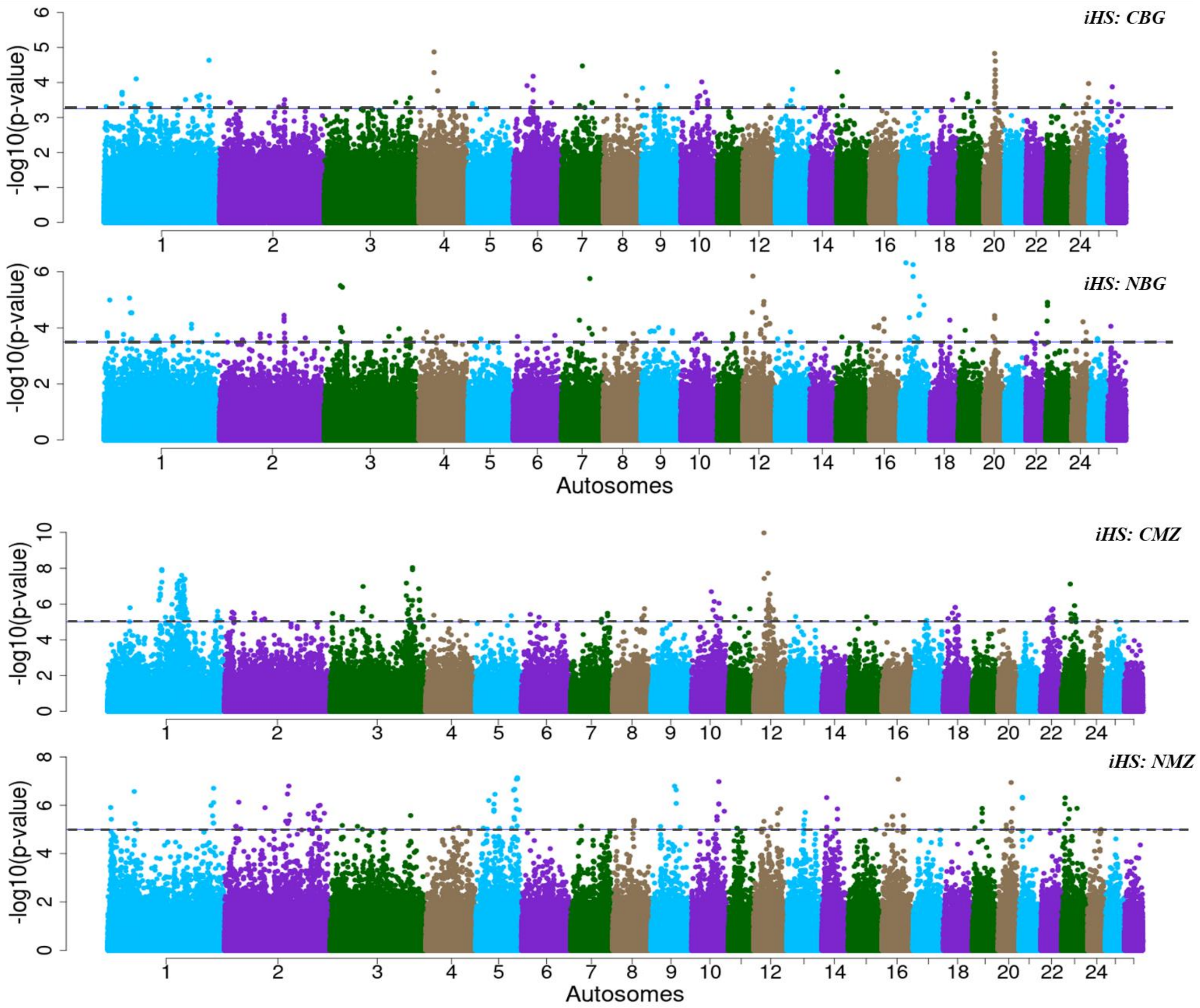

Supplement: Supplementary file 1 — Figures S1–S4 [file AGE-53-447-s002.pdf]
