# Supplementary material for: Genomic analysis of 10 years of artificial selection in community‐based breeding programs in two Ethiopian indigenous sheep breeds
Source: Anim Genet. 2022 Apr 15;53(3):447–51. doi: 10.1111/age.13190 (PMC10138745; doi:10.1111/age.13190)
Supplement: Supplementary file 2 — Table S1–S3 [file AGE-53-447-s001.docx]

Supplementary Table S1. Candidate regions that overlapped between at least two methods of selection signature analysis in Bonga and Menz sheep.

| ***Breed*** | ***Reg*** | ***OAR*** | ***Start****** | ***Stop****** | ***Size (Mb)*** | ***Method*** | | | | | ***Top SNP (LR-GWAS)*** | ***No. of genes*** | ***Genes (Top gene**)*** | ***P_value**** |
| --- | --- | --- | --- | --- | --- | --- | --- | --- | --- | --- | --- | --- | --- | --- |
|  |  |  |  |  |  | ***ROH*** | ***iHS*** | ***LR*** | ***FST*** | ***XP_EHH*** |  |  |  |  |
| *BONGA*  *(CBG vs NBG)* | 1 | 1 | 2800794 | 3000794 | 0.200 | 🗶 | 🗶 | ✓ | ✓ | 🗶 | *oar3_OAR1_2900794* | 6 | *TRAF3IP1, PER2,* ***ILKAP****, KLHL30, HES6, ERFE* | *4.2E-06* |
|  | 2 | 2 | 153570001 | 153810000 | 0.240 | 🗶 | ✓ | 🗶 | 🗶 | ✓ | oar3_OAR2_153730874 | 1 | *KCNJ3* | 7.0E-07 |
|  | 3 | 2 | 39732580 | 39880000 | 0.147 | 🗶 | 🗶 | ✓ | ✓ | 🗶 | oar3_OAR2_39851345 | 1 | *CDCA2* | 5.9E-06 |
|  | 4 | 4 | 73262692 | 73462692 | 0.200 | 🗶 | 🗶 | ✓ | ✓ | 🗶 | oar3_OAR4_73362692 | 1 | *ZNF804B* | 4.3E-06 |
|  | 5 | 8 | 83160001 | 83380000 | 0.220 | 🗶 | ✓ | 🗶 | ✓ | 🗶 | oar3_OAR8_83251261 | 3 | *SLC22A,* ***PLG****, MAP3K4* | 8.7E-07 |
| 🗶🗶MENZ  (CMZ vs NMZ) | 1 | 1 | 53260001 | 53511746 | 0.252 | 🗶 | ✓ | ✓ | 🗶 | 🗶 | oar3_OAR1_53411746 | 2 | *AK5, ZZZ3* | 3.9E-06 |
|  | 2 | 2 | 153226139 | 153319604 | 0.093 | ✓ | 🗶 | 🗶 | ✓ | 🗶 | _ | 2 | *KCNJ3 (Forwards), NR4A2(Backwards)* | _ |
|  | 3 | 3 | 50008278 | 50208278 | 0.200 | 🗶 | 🗶 | ✓ | ✓ | 🗶 | OAR3_52702802.1 | 1 | *LOC654331 (pancreatitis-associated protein I)* | 3.9E-06 |
|  | 4 | 3 | 207550001 | 207710000 | 0.160 | 🗶 | 🗶 | ✓ | ✓ | 🗶 | oar3_OAR3_207645351 | 10 | *CDCA3, GNB3, P3H3, GPR162, CD4, LAG3,* ***MLF2****, COPS7A, PIANP, ZNF384* | 5.2E-06 |
|  | 5 | 10 | 49776312 | 49976312 | 0.200 | 🗶 | 🗶 | ✓ | 🗶 | ✓ | oar3_OAR10_49876312 | 1 | *KLF12 (Backwards),* | 3.8E-06 |
|  | 6 | 10 | 79026631 | 79226631 | 0.200 | 🗶 | 🗶 | ✓ | ✓ | 🗶 | oar3_OAR10_79126631 | 2 | *LOC105616238 (Backwards), LOC105607422 (Forwards)* | 3.9E-06 |
|  | 7 | 12 | 51330001 | 51646452 | 0.316 | 🗶 | ✓ | ✓ | 🗶 | 🗶 | oar3_OAR12_51546452 | 1 | *LOC105616544* | 4.0E-06 |
|  | 8 | 16 | 38977896 | 39260850 | 0.283 | 🗶 | 🗶 | ✓ | ✓ | 🗶 | oar3_OAR16_39137420 | 7 | *PR, AGXT2,* ***DNAJC21****, BRIX1, RAD1, TTC23L, RAI14* | 6.4E-06 |
|  | 9 | 16 | 69432333 | 69632333 | 0.200 | 🗶 | 🗶 | ✓ | ✓ | 🗶 | s10403.1 | 1 | *LOC105602652* | 3.9E-06 |
|  | 10 | 21 | 31635634 | 31914251 | 0.279 | 🗶 | 🗶 | ✓ | ✓ | 🗶 | oar3_OAR21_31806097 | 2 | ***ARHGAP32****, BARX2* | 5.3E-06 |
|  | 11 | 25 | 21967608 | 22243113 | 0.276 | 🗶 | 🗶 | ✓ | ✓ | 🗶 | OAR25_23045938.1 | 1 | *CTNNA3* | 5.2E-06 |

*Significant markers following LR-GWAS Bonferroni correction P < 0.001; ** Top genes around the top marker based on LR-GWAS are in bold; ***The positions shown in the Start and Stop columns are referenced to the Oar_v3.1 sheep genome build.

Supplementary Table S2a. The topmost Enriched functional term clusters and their enrichment scores following DAVID 6.9 analysis for genes identified by all methodologies (249 genes) when Bonga CBBP individuals were contrasted with their non-CBBP counterparts.

| ***Category*** | ***ID*** | ***Term*** | ***Gene Count**** | % ** | ***P-value*** | ***Genes*** | ***Benjamini****** |
| --- | --- | --- | --- | --- | --- | --- | --- |
| GOTERM_MF | GO:0008270 | Zinc ion binding | 22 | 9.016 | 0.0143 | *TRIM40, TRIM52, TRIM41, USP3, NANOS3, JADE1, LTN1, PHF7, THAP1, NR4A1, ZNRD1, MAN2A2, XIRP2, TRIM26, TRIM15, ZSWIM4, MNAT1, AMZ1, SQSTM1, TRIM10, CPA6, TAF2* | 0.900 |
| GOTERM_CC | GO:0005622 | Intracellular | 16 | 6.557 | 0.0180 | *DFFA, TRIM52, ZNF382, DIRAS2, DDX43, ZNF45, HTR2A, SYTL3, LAT2, ZFP57, GNA12, IFT27, ZNF404, TRIM15, DOCK1, ZNF189* | 0.953 |
| GOTERM_MF | GO:0003676 | Nucleic acid binding | 14 | 5.738 | 0.0237 | *N6AMT1, PIWIL4, ZNF382, ZBTB26, GPATCH2, ZNF45, ZBTB6, ZNRD1, ZFP57, SYNJ1, ZSCAN23, ENPP1, ZNF404, ZNF189* | 0.978 |
| GOTERM_MF | GO:0004869 | Cysteine-type endopeptidase inhibitor activity | 3 | 1.230 | 0.0330 | *CST3, CST11, CSTL1* | 0.995 |
| GOTERM_CC | GO:0031901 | Early endosome membrane | 3 | 1.230 | 0.0363 | *SNX19, VPS33B, EPHB1* | 0.998 |
| GOTERM_BP | GO:0045087 | Innate immune response | 5 | 2.049 | 0.0374 | *TRIM26, TRIM15, CD1D, CLEC4E, TRIM10* | 1.000 |
| GOTERM_CC | GO:0045211 | Postsynaptic membrane | 4 | 1.639 | 0.0407 | *GABBR1, DLG2, CHRNB3, CHRNA6* | 0.999 |
| GOTERM_BP | GO:0030036 | Actin cytoskeleton organization | 4 | 1.639 | 0.0428 | *NISCH, NUAK2, DIAPH3, XIRP2* | 1.000 |
| GOTERM_CC | GO:0048471 | Perinuclear region of cytoplasm | 9 | 3.689 | 0.0447 | *ARFGEF1, PPP1R16B, TF, KIRREL, VAPA, NANOS3, VPS33B, RACK1, PICALM* | 1.000 |

* Number of genes involved in the term; **The proportion of genes involved in the term out of the total number of 249 genes identified when Bonga CBBP are contrasted with Bonga non-CBBP; ***Modified fisher exact P-Value (EASE Score), where the smaller the more enriched. A modified Fisher Exact P-value (<https://david.ncifcrf.gov/content.jsp?file=functional_annotation.html>).

Supplementary Table S2b. The topmost Enriched functional term clusters and their enrichment scores following DAVID analysis for genes identified by all methodologies (390 genes) when Menz CBBP individuals were contrasted with their non-CBBP counterparts.

| ***Category*** | ***ID*** | ***Term*** | ***Gene Count**** | ***%***** | ***P-value*** | ***Genes*** | ***Benjamini****** |
| --- | --- | --- | --- | --- | --- | --- | --- |
| GOTERM_CC | GO:0005882 | Intermediate filament | 6 | 1.596 | 0.0046 | *KRT39, PKP2, KRT23, MYO5A, KRT20, KRT40* | 0.655 |
| GOTERM_BP | GO:0006182 | cGMP biosynthetic process | 3 | 0.798 | 0.0072 | *NPPB, NPPA, AQP1* | 0.999 |
| KEGG | oas04724 | Glutamatergic synapse | 7 | 1.862 | 0.0086 | *PLA2G4F, PLA2G4D, PLA2G4E, HOMER2, GRM6, GNB3, GRIK1* | 0.800 |
| GOTERM_CC | GO:0005903 | Brush border | 5 | 1.330 | 0.0118 | *MYO1D, ACTN3, MYH9, DNM1L, AQP1* | 0.933 |
| GOTERM_MF | GO:0003774 | Motor activity | 4 | 1.064 | 0.0132 | *MYO1D, MYH15, MYO5C, MYO5A* | 0.957 |
| GOTERM_BP | GO:0009395 | Phospholipid catabolic process | 3 | 0.798 | 0.0247 | *PLA2G4F, PLA2G4D, PLA2G4E* | 1.000 |
| GOTERM_CC | GO:0005730 | Nucleolus | 19 | 5.053 | 0.0291 | *NOP56, MED1, USP36, HOMER2, HN1, URB1, NOL4, MIF4GD, GTPBP4, TXN2, GCFC2, CRACR2A, ZZZ3, GRB2, BRIX1, CDK12, ERGIC2, PAFAH1B2, RPL19* | 0.999 |
| GOTERM_CC | GO:0016459 | Myosin complex | 4 | 1.064 | 0.0305 | *MYO1D, MYH15, MYO5C, MYO5A* | 0.999 |
| GOTERM_CC | GO:0005737 | Cytoplasm | 58 | 15.426 | 0.0308 | *TTC25, TNFAIP3, KRT20, YARS2, MYLK, AQP1, AKAP12, NPPA, XPO5, DHX58, CEP97, POLH, UPF2, CD96, PLA2G4D, DESI2, PLA2G4E, ARMC7, DNTT, MIF4GD, ADRA2A, LATS1, ACLY, BDH2, DNAJC7, AMPH, UTRN, MAPRE2, ERGIC2, PAFAH1B2, TELO2, CNP, KLHL10, COPS7A, AK5, GTF2E1, DPP3, KIAA1524, HMBOX1, KATNA1, VPS16, HN1L, HOMER2, MAD2L1BP, ELP2, DCDC2, SNCAIP, PLXDC1, CENPE, CCS, KITLG, WNK1, ETNK1, SP4, INTS9, PPIL4, DZIP3, TUBA8* | 0.999 |
| GOTERM_BP | GO:0043123 | Positive regulation of I-kappaB kinase/NF-kappaB signaling | 7 | 1.862 | 0.0391 | *NDFIP2, CANT1, TAB2, FASLG, TRIM38, LTBR, TNFRSF1A* | 1.000 |

* Number of genes involved in the term; **The proportion of genes involved in the term to the total number of 390 genes identified in Menz sheep; ***Modified fisher exact P-Value (EASE Score), where the smaller the more enriched.

Supplementary Table S3a. QTLs spanning the candidate regions that overlapped between at least two methods of detecting selection signatures in Bonga sheep

| **OAR** | **Region span (Mbp)*** | **QTL trait** | **QTL type** | **QTL symbol** | **QTL span (Mbp)** | **QTL ID** |
| --- | --- | --- | --- | --- | --- | --- |
| 1 | 2.80- 3.00 | No QTL Present |  |  |  |  |
| 2 | 153.6 – 153.8 | Meat arachidonic acid content | Meat and Carcass | FA-C20:4 | 2.20-206.20 | 17213 |
|  |  | Meat docosapentaenoic acid content | Meat and Carcass | FA-C22:5 | 6.94-234.59 | 17217 |
|  |  | Meat color L* | Meat and Carcass | CIEL | 92.53-172.20 | 14163 |
|  |  | Meat color L* | Meat and Carcass | CIEL | 81.46-158.52 | 14167 |
|  |  | Milk protein percentage | Milk protein | PP | 32.02-207.42 | 57738 |
|  |  | Milk fat percentage | Milk fat | MF | 88.05-248.91 | 13915 |
|  |  | Milk lactose yield | Milk composition | MLACT | 129.31-203.51 | 13991 |
|  |  | Meat color b* | Meat and Carcass | CIELB | 83.31-172.20 | 14165 |
|  |  | Meat color a* | Meat and Carcass | CIELA | 77.44-172.20 | 14164 |
|  |  | Milk Yield | Milk yield | MY | 143.86-204.38 | 13992 |
|  |  | Ultimate pH | Meat and Carcass | UPH | 77.44-172.20 | 14162 |
|  |  | Shear force | Meat and Carcass | SF | 91.74-172.20 | 14166 |
|  |  | Meat color a* | Meat and Carcass | CIELA | 83.31-172.20 | 14168 |
|  |  | Meat color b* | Meat and Carcass | CIELB | 83.31-172.20 | 14169 |
|  |  | Meat eicosapentaenoic acid content | Meat and Carcass | FA-C20:5 | 6.94-238.10 | 17214 |
|  |  | Hot carcass weight | Meat and Carcass | HCWT | 55.01-247.09 | 14279 |
|  |  | Body weight (slaughter) | Production/Growth/Body weight | BW | 26.50-246.14 | 14280 |
|  |  | Meat linolenic acid content | Meat and Carcass | FA-C18:3 | 6.94-238.10 | 17210 |
| 2 | 39.73- 39.88 | Rump width | Exterior/Conformation | RUMPW | 25.94-50.37 | 14179 |
|  |  | Meat arachidonic acid content | Meat and Carcass | FA-C20:4 | 2.20-206.20 | 17213 |
|  |  | Meat docosapentaenoic acid content | Meat and Carcass | FA-C22:5 | 6.94-234.60 | 17217 |
|  |  | Milk protein percentage | Milk/Milk Protein | PP | 32.02-207.42 | 57738 |
|  |  | Milk fat percentage | Milk/Milk Fat | MF | 8.80-248.90 | 13915 |
|  |  | Meat eicosapentaenoic acid content | Meat and Carcass | FA-C20:5 | 6.94-238.10 | 17214 |
|  |  | Body weight (slaughter) | Production/Growth/Body weight | BW | 26.50-246.14 | 14280 |
|  |  | Meat linolenic acid content | Meat and Carcass | FA-C18:3 | 6.94-238.10 | 17210 |
| 4 | 73.26-73.46 | Primary fiber diameter coefficient of variance | Wool/Fibre | CVFD_PRI | 6.80-115.07 | 14019 |
| 8 | 83.16-83.38 | Body weight (birth) | Production/Growth/Body weight | BIRTH_WT | 78.54-87.36 | 13793 |
|  |  | Milk lactose yield | Milk/Milk Composition | MLACT | 79.77-87.36 | 16014 |
|  |  | Milk fat percentage | Milk/Milk Fat | MF | 60.41-90.70 | 13999 |
|  |  | Trichostrongylus adult and larva count | Health/Parasite Resistance | LATRICH_2 | 3.06-87.36 | 12899 |
|  |  | Trichostrongylus adult and larva count | Health/Parasite Resistance | LATRICH_2 | 3.06-87.36 | 12900 |
|  |  | Fecal egg count | Health/Parasite Resistance | FECGEN | 62.54-87.36 | 16025 |

*Region span is based on the coordinates of Oar_v3.1 sheep genome build.

Supplementary Table S3b. QTLs spanning the candidate regions that overlapped between at least two methods of detecting selection signatures in Menz sheep

| **OAR** | **Region span (Mbp)** | **QTL trait** | **QTL type** | **QTL symbol** | **QTL span (Mbp)** | **QTL ID** |
| --- | --- | --- | --- | --- | --- | --- |
| 1 | 53.26- 53.51 | Meat docosapentaenoic acid content | Meat and Carcass | FA-C22:5 | 38.33-249.07 | 17216 |
|  |  | Bone weight in carcass | Meat and Carcass/Anatomy | BONE_WT | 33.73-239.52 | 14275 |
|  |  | Muscle weight in carcass | Meat and Carcass/Anatomy | MUSWT | 30.77-184.64 | 14276 |
|  |  | Lean meat yield percentage | Meat and Carcass/Anatomy | LMYP | 9.43-197.49 | 14278 |
|  |  | Carcass fat percentage | Meat and Carcass/Fatness | FATP | 10.48-266.47 | 14277 |
|  |  | Carcass bone percentage | Meat and Carcass/Anatomy | BONEP | 39.01-62.67 | 14321 |
|  |  | Body weight (8 weeks) | Production/Growth/Body weight | BW | 40.96-86.43 | 13885 |
|  |  | Meat eicosapentaenoic acid content | Meat and Carcass/Fatty Acid Content | FA-C20:5 | 38.33-242.14 | 17215 |
|  |  | Meat polyunsaturated fatty acid content | Meat and Carcass/Fatty Acid Content | PUFA | 43.89-249.07 | 17221 |
| 2 | 153.22-153.31 | Meat color L* | Meat and Carcass/Meat Colour | CIEL | 92.53-172.20 | 14163 |
|  |  | Meat color L* | Meat and Carcass/Meat Colour | CIEL | 81.46-158.52 | 14167 |
|  |  | Milk protein percentage | Milk/Milk Protein | PP | 32.02-207.42 | 57738 |
|  |  | Milk fat percentage | Milk/Milk Fat | MF | 8.80-248.90 | 13915 |
|  |  | Milk lactose yield | Milk/Milk Composition | MLACT | 129.31-203.51 | 13991 |
|  |  | Meat color b* | Meat and Carcass/Meat Colour | CIELB | 83.31-172.20 | 14165 |
|  |  | Meat color a* | Meat and Carcass/Meat Colour | CIELA | 77.44-172.20 | 14164 |
|  |  | Milk Yield | Milk/Milk Yield | MY | 143.86-204.38 | 13992 |
|  |  | Meat arachidonic acid content | Meat and Carcass/Fatty Acid Content | FA-C20:4 | 2.20-206.20 | 17213 |
|  |  | Ultimate pH | Meat and Carcass/Chemistry | UPH | 77.44-172.20 | 14162 |
|  |  | Shear force | Meat and Carcass/Meat Texture | SF | 91.74-172.20 | 14166 |
|  |  | Meat color a* | Meat and Carcass/Meat Colour | CIELA | 83.31-172.20 | 14168 |
|  |  | Meat color b* | Meat and Carcass/Meat Colour | CIELB | 83.31-172.20 | 14169 |
|  |  | Meat eicosapentaenoic acid content | Meat and Carcass/Fatty Acid Content | FA-C20:5 | 6.94-238.10 | 17214 |
|  |  | Hot carcass weight | Meat and Carcass/Anatomy | HCWT | 55.01-247.09 | 14279 |
|  |  | Body weight (slaughter) | Production/Growth/Body weight | BW | 26.50-246.14 | 14280 |
|  |  | Meat linolenic acid content | Meat and Carcass/Fatty Acid Content | FA-C18:3 | 6.94-238.10 | 17210 |
|  |  | Meat docosapentaenoic acid content | Meat and carcass/Fatty Acid Content | FA-C22:5 | 6.94-234.59 | 17217 |
| 3 | 50.00-50.20 | Internal fat amount | Meat and Carcass/Fatness | INTFAT | 42.04-188.72 | 14281 |
|  |  | Staple length | Wool/Fleece | SL | 1.18-224.28 | 14014 |
|  |  | Meat conjugated linoleic acid content | Meat and Carcass/Fatty Acid Content | MCLA | 49.22-156.72 | 17220 |
|  |  | Body weight (56 weeks) | Production/Growth/Body weight | BW | 14.08-74.63 | 13927 |
|  |  | Milk fat percentage | Milk/Milk Fat | MF | 38.62-75.52 | 13993 |
|  |  | Milk fat yield | Milk/Milk Fat | MFY | 42.91-59.86 | 13994 |
|  |  | Milk lactose yield | Milk/Milk Composition | MLACT | 39.00-72.11 | 13995 |
|  |  | Milk Yield | Milk/Milk Yield | MY | 41.37-76.86 | 13996 |
|  |  | Milk protein yield | Milk/Milk Protein | PY | 39.00-60.52 | 13997 |
| 3 | 207.55-207.71 | Staple length | Wool/Fleece | SL | 1.18-224.28 | 14014 |
|  |  | Reproductive seasonality | Reproduction/Fertility | ASREP | 190.63-208.10 | 16605 |
|  |  | Nematodirus FEC | Health/Parasite Resistance | NFEC | 190.93-208.10 | 12882 |
|  |  | Reproductive seasonality | Reproduction/Fertility | ASREP | 190.63-208.10 | 16604 |
|  |  | Fecal egg count | Health/Parasite Resistance | FECGEN | 201.52-212.35 | 16023 |
|  |  | Fecal oocyst count | Health/Parasite Resistance | FOC | 201.52-213.60 | 12905 |
| 10 | 49.78-49.98 | Fecal egg count | Health/Parasite Resistance | FECGEN | 24.22-86.44 | 13989 |
|  |  | Testes weight | Reproduction/Reproductive organs | TESTWT | 28.69-74.71 | 12923 |
|  |  | Fat weight in carcass | Meat and Carcass/Fatness | FATWT | 13.90-78.60 | 14292 |
|  |  | Carcass bone percentage | Meat and Carcass/Anatomy | BONEP | 13.90-78.60 | 14293 |
|  |  | Carcass fat percentage | Meat and Carcass/Fatness | FATP | 13.90-78.60 | 14294 |
|  |  | Lean meat yield percentage | Meat and Carcass/Anatomy | LMYP | 13.90-78.60 | 14295 |
| 12 | 51.33- 51.64 | No QTL present |  |  |  |  |
| 16 | 38.98-39.26 | Body weight (slaughter) | Production/Growth/Body weight | BW | 1.00-53.54 | 14306 |
|  |  | Dressing percentage | Meat and Carcass/Anatomy | DRESSING | 2.35-67.65 | 14305 |
|  |  | Subcutaneous fat area | Meat and Carcass/Fatness | SCFA | 22.23-40.49 | 14273 |
|  |  | Subcutaneous fat thickness | Meat and Carcass/Fatness | SCFT | 4.47-62.75 | 14309 |
|  |  | Subcutaneous fat area | Meat and Carcass/Fatness | SCFA | 4.47-60.23 | 14308 |
|  |  | Rear leg set | Exterior/Conformation | RLEGS | 36.16-39.50 | 14182 |
|  |  | Milk lactose content | Milk/Milk Composition | MLC | 39.00-39.00 | 213374 |
|  |  | Somatic Cell Score | Health/Mastitis | SCS | 39.00-39.00 | 213375 |
|  |  | Rennet coagulation time | Milk/Milk Processing | RCT | 39.00-39.00 | 213376 |
|  |  | Curd firming time | Milk/Milk Processing | TCURDF | 39.00-39.00 | 213377 |
|  |  | Lean meat yield percentage | Meat and Carcass/Anatomy | LMYP | 10.07-62.83 | 14307 |
| 21 | 31.63-31.91 | Meat docosapentaenoic acid content | Meat and Carcass/Fatty Acid Content | FA-C22:5 | 4.51-50.07 | 17218 |
|  |  | Average daily gain | Production/Growth | ADG | 15.66-43.69 | 13946 |
|  |  | Body weight (56 weeks) | Production/Growth | BW | 15.66-43.69 | 13930 |
|  |  | Average daily gain | Production/Growth | ADG | 15.66-43.69 | 13952 |
|  |  | Meat gadoleic acid content | Meat and Carcass/Fatty Acid Content | FA-C20:1 | 4.51-50.07 | 17207 |
|  |  | Body weight (43 weeks) | Production/Growth | BW | 15.66-43.69 | 13925 |
|  |  | Body weight (83 weeks) | Production/Growth | BW | 15.66-43.69 | 13936 |
|  |  | Average daily gain | Production/Growth | ADG | 15.66-43.69 | 13959 |
|  |  | Fecal egg count | Health/Parasite Resistance | FECGEN | 31.70-32.24 | 95636 |
|  |  | Milk protein percentage | Milk/Milk Protein | PP | 31.54-32.91 | 57734 |
|  |  | Salmonella abortusovis susceptibility | Health/Disease Susceptibility | SAOS | 23.63-42.89 | 17195 |
|  |  | Meat myristic acid content | Meat and Carcass/Fatty Acid Content | FA-C14:0 | 8.49-50.07 | 17201 |
|  |  | Meat palmitic acid content | Meat and Carcass/Fatty Acid Content | FA-C16:0 | 4.51-50.07 | 17202 |
|  |  | Meat linolenic acid content | Meat and Carcass/Fatty Acid Content | FA-C18:3 | 4.51-50.07 | 17211 |
|  |  | Salmonella abortusovis susceptibility | Health/Disease Susceptibility | SAOS | 28.89-42.89 | 17196 |
|  |  | Meat stearic acid content | Meat and Carcass/Fatty Acid Content | FA-C18:0 | 4.51-50.07 | 17203 |
|  |  | Meat oleic acid content | Meat and Carcass/Fatty Acid Content | FA-C18:1 | 4.51-50.07 | 17205 |
|  |  | Meat cis-vaccenic acid content | Meat and Carcass/Fatty Acid Content | FA-C18:1 | 4.51-50.07 | 17206 |
|  |  | Meat linoleic acid content | Meat and Carcass/Fatty Acid Content | FA-C18:2 | 4.51-50.07 | 17209 |
|  |  | Meat arachidonic acid content | Meat and Carcass/Fatty Acid Content | FA-C20:4 | 4.51-50.00 | 17212 |
| 25 | 21.97-22.24 | Testes weight | Reproduction/Reproductive Organs | TESTWT | 1.56-45.37 | 12925 |
|  |  | Staple length | Wool/Fleece | SL | 1.56-41.81 | 14016 |
|  |  | Mean fiber diameter | Wool/Fibre | MFDIAM | 1.56-41.81 | 14018 |
|  |  | Primary fiber diameter coefficient of variance | Wool/Fibre | CVFD_PRI | 1.56-41.81 | 14021 |

*Region span is based on the coordinates of Oar_v3.1 sheep genome build.
